# Comparison of Effectiveness of High Dose Statin Monotherapy With Combination of Statin and Ezetimibe to Prevent Cardiovascular Events in Patients With Acute Coronary Syndrome: A Systematic Review and Meta-Analysis

**DOI:** 10.7759/cureus.55922

**Published:** 2024-03-10

**Authors:** Nanush Damarpally, Tanya Sinha, Michelle Maricela Nunez, Manisha Guntha, Thin M Soe, Sandipkumar S Chaudhari, Roba A Ibrahim, Shamsha Hirani

**Affiliations:** 1 Medicine, Houston Community College, Houston, USA; 2 Medicine, Tribhuvan University, Kirtipur, NPL; 3 Medicine, Universidad Autónoma de Guadalajara, Zapopan, MEX; 4 Internal Medicine, Sinai-Grace Hospital, Detroit, USA; 5 Medicine, University of Medicine (1) Yangon, Yangon, MMR; 6 Cardiothoracic Surgery, University of Alabama, Birmingham, USA; 7 Family Medicine, University of North Dakota School of Medicine and Health Sciences, Fargo, USA; 8 Internal Medicine, Elrazi University, Khartoum, SDN; 9 Cardiology, Baqai Hospital, Karachi, PAK

**Keywords:** systematic review and meta analysis, acute coronary syndrome, cardiovascular events, ezetimibe+statin, high-statin therapy

## Abstract

This meta-analysis aimed to compare the effectiveness of high statin monotherapy and a combination of statin and ezetimibe to prevent cardiovascular outcomes in patients with acute coronary syndrome (ACS). The study was conducted according to Preferred Reporting Items for Systematic Reviews and Meta-Analyses (PRISMA) guidelines. We conducted comprehensive searches across online databases, including MEDLINE/ PubMed, EMBASE, and the Web of Science, to find the relevant articles from the databases' inception to 10 Feb 2024. Outcomes assessed in the meta-analysis included major cardiovascular events (MACE), all-cause mortality, stroke, myocardial infarction, and unplanned revascularization. Data analysis was conducted utilizing RevMan Version 5.3.1. The comparison of outcomes between the two groups involved the calculation of risk ratios (RR) accompanied by 95% confidence intervals (CI) using either a random or fixed-effect model. Five studies were included in this meta-analysis, encompassing 48,668 patients. The pooled analysis showed that the risk of all-cause mortality was higher in patients receiving high statin monotherapy. However, no significant differences in MACE, myocardial infarction, stroke, and revascularization were reported. While acknowledging the limitations, including the lack of randomized controlled trials and the dominance of one study in the analysis, these findings underscore the importance of further research to clarify the comparative effectiveness of these treatment modalities in preventing cardiovascular outcomes in ACS patients.

## Introduction and background

Lipid-lowering therapy (LLT) stands as a pivotal component in the management of patients grappling with acute coronary syndrome (ACS) [1]. Statins recognized as 3-hydroxy-3-methylglutaryl-coenzyme A reductase inhibitors play a crucial role in reducing low-density lipoprotein cholesterol (LDL-C) levels, thereby mitigating subsequent cardiovascular events [1]. Apart from their cholesterol-lowering effects, statins exhibit favorable anti-inflammatory properties, commonly referred to as pleiotropic effects, particularly beneficial for ACS patients [1-2]. Despite their efficacy, statins are associated with subjective myalgia, often termed a nocebo effect, affecting up to 30% of patients [1]. This phenomenon may necessitate dose adjustments, switching to alternative therapies, or supplementing with nonstatin LLT to mitigate the risk of therapy discontinuation, potentially impacting cardiovascular outcomes [3].

The 2019 European Society of Cardiology/European Atherosclerosis Society guidelines categorize post-ACS patients as very high-risk individuals, setting LDL-C reduction goals at <55mg/dL (1.4mmol/L) [4]. According to these guidelines, the recommended approach involves initiating monotherapy, with the addition of other LLT, if initial efforts prove inadequate (after 4-6 weeks) [4]. Notably, combination therapy with ezetimibe (class IIa) is suggested, followed by the addition of a PCSK9 inhibitor if targets remain unmet (class I) [4]. Ezetimibe, a novel cholesterol absorption inhibitor, effectively impedes cholesterol absorption by hindering the transit of dietary and biliary cholesterol across the intestinal wall [5].

Following the IMPROVE-IT trial, which showcased the superior efficacy of combined moderate-intensity statin and ezetimibe therapy compared to moderate-intensity statin monotherapy in hospitalized ACS patients [6], current guidelines advocate for the addition of ezetimibe therapy to maximize tolerated statin doses if LDL-C goals are not achieved [7]. However, clinical evidence supporting the notion that simvastatin-ezetimibe may serve as an alternative to high-intensity statin therapy in acute myocardial infarction (AMI) patients, particularly those at high risk, remains insufficient. While Pauriah et al. indirectly indicated the superiority of high-intensity statins over simvastatin-ezetimibe in improving clinical outcomes post-AMI, data regarding high-risk patients and outcomes other than mortality were lacking [8]. Conversely, Chang et al. reported the benefits of simvastatin-ezetimibe over high-potency statins in diabetic patients, albeit not in the context of ACS patients, with limitations in defining high-intensity statin therapy [9].

Given the scarcity of studies comparing high-dose statin monotherapy with combination therapy of statin and ezetimibe in ACS patients, our meta-analysis aims to fill this gap by analyzing available data to assess the effectiveness of these treatment modalities in preventing cardiovascular events in this patient population.

## Review

Methodology

Search Strategy and Study Selection

Our search strategy aimed to identify published articles comparing the lipid-lowering efficacy of high-intensity statin (daily dose reducing LDL cholesterol by approximately 50%) versus low/moderate-intensity statin (daily dose reducing LDL cholesterol by < 50%) in combination with ezetimibe for patients with acute coronary syndrome (ACS). Initially, we conducted comprehensive searches across online databases, including MEDLINE/ PubMed, EMBASE, and the Web of Science Search terms encompassed combinations of relevant PubMed MeSH terms and associated text terms such as statins, hydroxymethylglutaryl-CoA reductase inhibitors, 3-hydroxy-3-methylglutaryl coenzyme A reductase inhibitors, ezetimibe, and acute coronary syndrome. Additionally, we scrutinized the bibliographies of retrieved articles and pertinent reviews to identify further eligible studies. Our search, unrestricted by publication date, concluded on 10 Feb 2024.

Two authors independently conducted the review and selection process for studies to be included in the systematic review. Inclusion criteria comprised: (1) randomized clinical trials or observational studies; (2) comparison of high-intensity statin versus low- or moderate-intensity statin plus ezetimibe; (3) inclusion of at least 50% ACS patients; and (4) assessment of required outcomes. Disagreements regarding article inclusion were resolved through discussion. If a trial was reported in multiple publications, data were extracted from the most comprehensive account, with other publications utilized for clarification. Studies with follow-up periods of less than 12 months, as well as reviews and editorials, were excluded. Table 1 presents the PICO (Population, intervention, comparison group, and outcome). The study adhered to Preferred Reporting Items for Systematic Reviews and Meta-Analyses (PRISMA) guidelines, with any discrepancies between the two authors during the search and study selection process resolved through discussion.

**Table 1:** PICO criteria used in study selection PICO: Population, intervention, comparison group and outcome

Category	Description
Population	Patients with age of 18 years or more and having acute coronary syndrome
Intervention	High-intensity statin
Comparison group	Low/moderate-intensity statin with ezetimibe
Outcomes	Major cardiovascular events (MACE), all-cause mortality, stroke, myocardial infarction, and unplanned revascularization.

Data Extraction and Outcomes Assessed

Thorough reviews of full-text articles were independently conducted by the two authors, with the following data extracted from each study: first author’s surname; year of publication; participant count; patient demographics (mean age, number of males, diabetes, hypertension and history of myocardial infarction; administered treatments; and assessed outcomes, including major cardiovascular events (MACE), all-cause mortality, stroke, myocardial infarction, and unplanned revascularization. Quality assessment of included studies was done using the New-Castle Ottawa Scale (NCOS) and Cochrane risk of bias assessment tool for observational studies and RCTs, respectively.

Data Synthesis and Analysis

Data analysis was conducted utilizing RevMan Version 5.4.1 (The Cochrane Collaboration, Oxford, United Kingdom). The comparison of outcomes between the two groups involved the calculation of risk ratios (RR) accompanied by 95% confidence intervals (CI) using either a random or fixed-effect model, contingent upon the heterogeneity among the study results. Statistical significance was determined by a p-value less than 0.05. Heterogeneity was assessed using the I-square (I²) statistic, with a threshold of less than 50% indicating low heterogeneity and the application of a fixed-effect model. Conversely, for I² values exceeding 50%, a random-effect model was employed to compute effect estimates. We were unable to assess publication bias as number of included studies was less than 10.

Results

**Figure 1:**
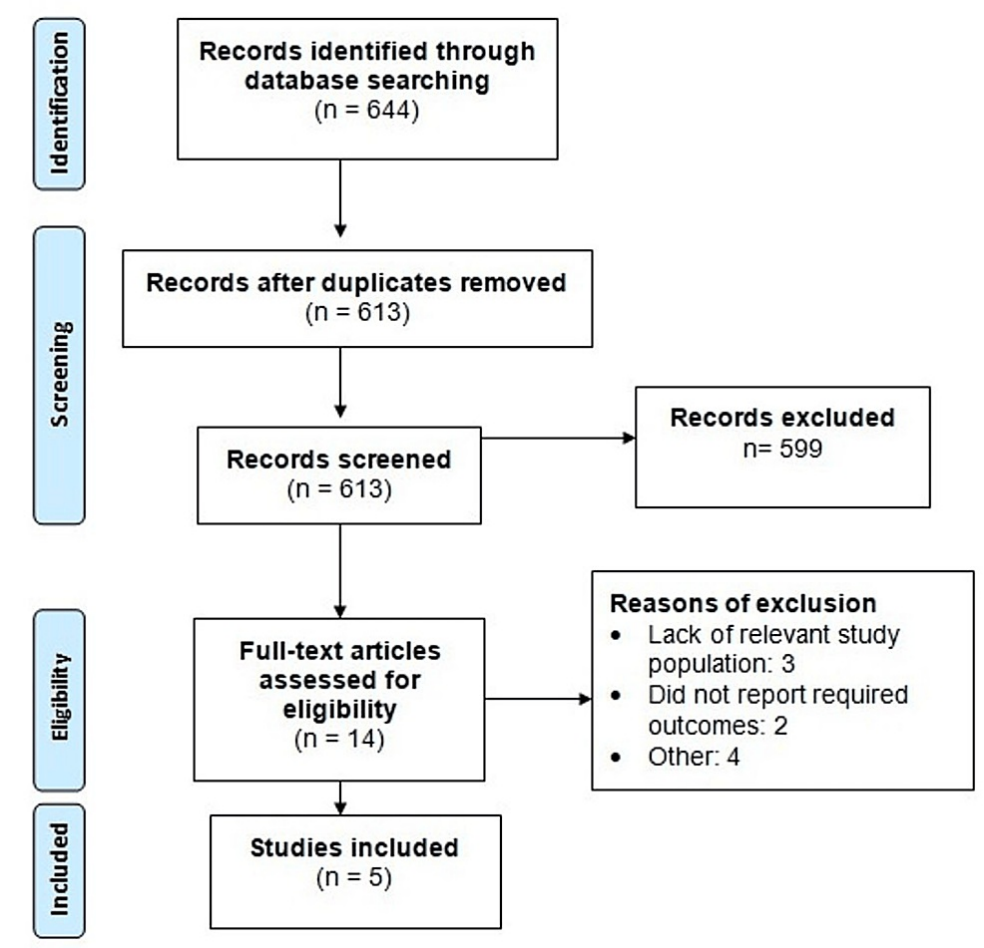
PRISMA flowchart of study selection

Utilizing online databases, a total of 644 studies were initially identified. After removing duplicates, a preliminary screening of 613 studies was conducted based on their abstracts and titles. After initial screening, 599 records were excluded as they did not meet the study objective or did not fulfill eligibility criteria. Subsequently, the full text of 14 studies was retrieved, and a detailed evaluation was undertaken according to predefined inclusion and exclusion criteria. Ultimately, five studies met the eligibility criteria and were included in the meta-analysis. The study selection process is depicted in (Figure 1), while (Table 2) provides an overview of the characteristics of the included studies. Notably, all included studies were observational, with a pooled analysis comprising 48,668 patients. Across these studies, the majority of patients were male. The quality assessment of the included studies (Table 3). Remarkably, all studies exhibited a high level of quality based on the assessment criteria.

**Table 2:** Characteristics of included studies NS: Not specified; MI: Myocardial infarction

Author	Year	Groups	Total Population (N)	Follow-up Durarion	Age (Years)	Males (n)	Hypertension (n)	Diabetes (n)	MI (n)
Jang et al. [10]	2024	Statin Monotherapy	10723	34 Months	NS	7590	9305	3516	4940
		Statin+ezetimibe	10723		NS	7544	9245	3546	4904
Ji et al. [11]	2016	Statin Monotherapy	671	12 Months	61.8	513	288	178	671
		Statin+ezetimibe	671		60.7	509	294	185	671
Kim et al. [12]	2021	Statin Monotherapy	4041	30 Months	59.8	3397	3611	1264	4041
		M.Statin+ezetimibe	233		59.1	190	208	80	233
Kim et al. [13]	2023	Statin Monotherapy	19148	12 Months	63	14254	12859	8622	9294
		M.Statin+ezetimibe	922		65	604	681	487	226
Lewek et al. [14]	2023	Statin Monotherapy	768	36 Months	64	509	594	217	188
		Statin+ezetimibe	768		63.5	512	580	228	192

**Table 3:** Quality assessment of included studies

Author	Selection	Comparison	Outcome	Overall
Jang et al. [10]	3	2	3	Good
Ji et al. [11]	4	1	3	Good
Kim et al. [12]	4	2	2	Good
Kim et al. [13]	3	2	3	Good
Lewek et al. [14]	3	1	2	Good

Major Cardiovascular Events (MACE)

**Figure 2:**
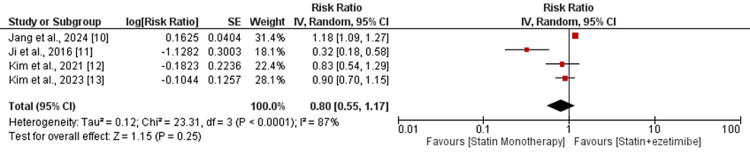
Comparing MACE between two groups MACE: major cardiovascular events Sources: References [10-13]

MACE was compared by four of the included studies. The pooled analysis found that there was no statistically significant difference in the risk of major adverse cardiovascular events (MACE) between statin monotherapy and statin-ezetimibe combination therapy. The risk ratio was 0.80 (95% CI 0.55, 1.17), with a p-value of 0.25, as shown (Figure 2). Significant heterogeneity existed between the studies (I-square: 87%; P < 0.0001).

Other Outcomes

The analysis revealed a risk ratio (RR) of all-cause mortality at 1.32 (95% CI: 1.18, 1.47), demonstrating a statistically significant p-value of 0.0001. This finding suggests a heightened risk of all-cause mortality in the statin monotherapy group when compared to the combination group. Conversely, the risk ratio for stroke was determined to be 0.93 (95% CI: 0.59, 1.47) with a p-value of 0.7762, indicating a lack of statistically significant difference in stroke risk between the two groups. Similarly, the risk ratio for myocardial infarction was calculated at 0.97 (95% CI: 0.84, 1.12) with a p-value of 0.6900, suggesting no statistically significant difference in myocardial infarction risk between the groups. Moreover, the risk ratio for revascularization stood at 0.63 (95% CI: 0.30, 1.33) with a p-value of 0.2384, signifying no statistically significant distinction in revascularization risk between the statin monotherapy and combination groups, as shown (Table 4).

**Table 4:** Comparison of outcomes between two groups RR: Risk ratio; CI: Confidence interval * Significant at p-value< 0.05

Outcomes	No. of Studies	RR (95% CI)	P-value	I-square
All-cause Mortality	5	1.32 (1.18 to 1.47)	0.0001*	24%
Stroke	3	0.93 (0.59 to 1.47)	0.77	62%
Myocardial Infarction	4	0.97 (0.84 to 1.12)	0.69	0%
Revascularization	3	0.63 (0.30 to 1.33)	0.23	84%

Discussion

This meta-analysis was conducted to assess the comparative efficacy of high-intensity statin monotherapy versus low or moderate-intensity statin combined with ezetimibe therapy in mitigating cardiovascular events among patients diagnosed with the acute coronary syndrome (ACS). Our pooled analysis revealed no statistically significant differences in the risk of major adverse cardiovascular events (MACE), cardiac death, revascularization, stroke, or myocardial infarction between the two treatment modalities. However, the combined analysis of data from five studies indicated a notably elevated risk of all-cause mortality among patients treated with high-intensity statin monotherapy in comparison to those receiving combination therapy. Overall, our meta-analysis incorporated findings from five studies, with the recent observational study conducted by Jang et al. [10] exerting the most substantial influence on our analysis due to its larger sample size and nearly three-year follow-up duration. Notably, this study highlighted the favorable impacts of combination therapy, demonstrating reductions in all-cause mortality, MACE, and stroke incidence. While all studies underscored a heightened risk of all-cause mortality associated with high-intensity statin monotherapy compared to combination therapy, only one study reported a statistically significant difference between the two groups [10]. This disparity may be attributed to the larger sample size of the aforementioned study, as opposed to the smaller sample sizes of the others, which may have lacked the statistical power to detect significant differences between treatment modalities.

The findings of our meta-analysis align with those of previous interventional and observational studies. For instance, a Chinese randomized clinical trial demonstrated the superior efficacy of moderate-intensity statin combined with ezetimibe therapy over high-intensity statin monotherapy in reducing low-density lipoprotein cholesterol levels among stable patients [15]. Similarly, observational studies involving patients receiving combination lipid-lowering therapy, with a sample size of fewer than 1000 patients, reported comparable effectiveness of both therapy options following acute myocardial infarction or percutaneous coronary intervention [12]. However, there is a notable absence of randomized controlled trials (RCTs) directly comparing these two therapeutic approaches in patients with acute coronary syndrome (ACS). Moreover, the recent RACING trial illustrated the non-inferiority of combination therapy compared to high-intensity statin monotherapy in patients with stable atherosclerotic cardiovascular disease. This trial demonstrated a higher control rate of low-density lipoprotein cholesterol levels and greater therapy adherence in the combination therapy group [16]. While this trial included subjects with various vascular disease conditions, it did not specify patients who underwent percutaneous coronary intervention. Despite the extensive exploration of high-intensity monotherapy and moderate-intensity combination treatment to reduce lipid levels for secondary prevention, there remains insufficient evidence to support the preemptive use of combination therapy in high-risk patients who have undergone stent implantation for acute coronary syndrome [6, 17]. Our meta-analysis contributes to this body of evidence by establishing the additional benefits of combining moderate-intensity statins with ezetimibe in patients with acute coronary syndrome.

Current clinical guidelines advocate for initiating and maintaining high-potency statins, which are capable of reducing low-density lipoprotein cholesterol levels by over 50%. Given that the benefits of statin therapy hinge largely on individual absolute risks, it is crucial to emphasize prolonged statin treatment in high-risk patients, such as those undergoing percutaneous coronary intervention [18]. However, the advancement in lipid management within routine clinical practice has progressed slower than anticipated. Research indicates that among Medicare beneficiaries hospitalized for myocardial infarction, less than two-thirds adhered to high-intensity statin therapy at 6 months (58.9%) and 2 years (41.6%) post-discharge [19]. Due to the high failure rate in achieving the low-density lipoprotein target among patients with atherosclerotic cardiovascular disease, the 2019 European Society of Cardiology guidelines advocate for the addition of ezetimibe therapy to the maximally tolerated intensity of statin treatment. This recommendation is supported by a meta-analysis of eighteen articles, which demonstrated that low to moderate-intensity statin plus ezetimibe therapy significantly reduced levels of LDL-C, triglycerides, and hs-CRP compared to high-intensity statin therapy.

Regarding safety, there were no significant differences between the two treatments regarding ALT elevation, but high-intensity statin therapy led to a significant increase in AST and CK compared to combination therapy [20]. Another study highlighted that adherence to moderate-intensity statins plus ezetimibe therapy may be higher compared to high-intensity statin therapy. This finding suggests that patients initially prescribed moderate-intensity statins plus ezetimibe were more likely to sustain their treatment regimen, primarily due to the de-escalation of initial therapy in patients initially receiving high-intensity statins [13].

The present meta-analysis does not identify any statistical differences concerning cardiovascular outcomes between the two treatment groups. However, it acknowledges that prior meta-analyses have demonstrated the significant impact of combination therapy on lipid profiles and other biomarkers [20]. Essentially, while the current study does not observe a statistical advantage, it acknowledges the existing evidence from previous research suggesting benefits in terms of lipid profile and other biomarkers associated with combination therapy. Taken together, these findings advocate for a pragmatic clinical trial involving patients with ACS, ensuring adequate representation of racial and ethnic groups, to evaluate the impact of combining low to moderate-intensity statin with ezetimibe versus high-dose statin monotherapy.

The current meta-analysis is subject to several limitations that warrant acknowledgment. Firstly, a notable limitation is the absence of randomized controlled trials (RCTs) directly comparing cardiovascular events between high-dose statin monotherapy and combination therapy. The observational nature of the studies included in this analysis introduces inherent biases, such as the lack of control over confounding variables, which may impact the robustness of the findings. Future studies are therefore needed to comprehensively assess the effects of these treatment modalities on the risk of cardiovascular outcomes. Secondly, it is important to note that the study conducted by Jang et al. [10] significantly contributed to the overall weight of each outcome analyzed in this meta-analysis.

Consequently, relying on a single study for a substantial portion of the data may potentially skew the results. Furthermore, the majority of the other studies included in the analysis had limited sample sizes, which could affect the generalizability and reliability of the findings. Therefore, caution should be exercised when interpreting the results, and additional research with larger sample sizes is warranted to corroborate these findings and provide a more comprehensive understanding of the comparative effectiveness of these treatment options.

## Conclusions

In conclusion, our meta-analysis found no statistically significant difference in major adverse cardiovascular events (MACE), stroke, myocardial infarction, or revascularization between high-intensity statin monotherapy and low or moderate-intensity statin plus ezetimibe combination therapy in patients with acute coronary syndrome (ACS). However, a notable increase in the risk of all-cause mortality was observed with high-intensity statin monotherapy. While acknowledging the limitations, including the lack of randomized controlled trials and the dominance of one study in the analysis, these findings underscore the importance of further research to clarify the comparative effectiveness of these treatment modalities in preventing cardiovascular outcomes in ACS patients.
